# Photon Absorption Remote Sensing Virtual Histopathology: A Preliminary Exploration of Diagnostic Equivalence to Gold-Standard H&E Staining in Skin Cancer Excisional Biopsies

**DOI:** 10.3390/diagnostics16203315

**Published:** 2026-10-12

**Authors:** Benjamin R. Ecclestone, James E. D. Tweel, Marie Abi Daoud, Hager Gaouda, Deepak Dinakaran, Michael P. Wallace, Ally Khan Somani, Gilbert Bigras, John R. Mackey, Parsin Haji Reza

**Affiliations:** 1 System Design Engineering, University of Waterloo, 200 University Ave W, Waterloo, ON N2L 3G1, Canada; james.tweel@uwaterloo.ca (J.E.D.T.); michael.wallace@uwaterloo.ca (M.P.W.); 2 Department of Pathology & Laboratory Medicine, University of Calgary, 2500 University Dr NW, Calgary, AB T2N 1N4, Canada; marie.abidaoud@albertahealthservices.ca; 3 Medical Biophysics, University of Toronto, 27 King’s College Cir, Toronto, ON M5S 1A1, Canada; deepak.dinakaran@sunnybrook.ca; 4 Department of Dermatology, Indiana University School of Medicine, 550 N. University Blvd., UH 3240, Indianapolis, IN 46202, USA; 5 SkinMD LLC, 16105 S. LaGrange Road, Orland Park, IL 60467, USA; 6 Department of Laboratory Medicine, University of Alberta, 116 St & 85 Ave, Edmonton, AB T6G 2R3, Canadajohn.mackey@illumisonics.com (J.R.M.); 7 illumiSonics Inc., 22 King St S Suite #300, Waterloo, ON N2J 1N8, Canada

**Keywords:** virtual histology, label-free, absorption microscopy, PARS, virtual staining, deep learning, artificial intelligence

## Abstract

**Background:** Photon Absorption Remote Sensing (PARS) enables label-free imaging of subcellular morphology by observing absorption interactions. Coupled with deep-learning, PARS produces label-free virtual hematoxylin and eosin (H&E) images in unprocessed tissues. This study evaluates the diagnostic performance of PARS-derived virtual H&E images in skin excisions, including squamous cell carcinoma (SCC), basal cell carcinoma (BCC), and normal skin. **Methods:** Sixteen unstained formalin-fixed paraffin-embedded excisions were PARS-imaged, then stained with chemical H&E dye. Seven fellowship-trained dermatopathologists assessed all 32 images (16 PARS and 16 H&E) in a masked randomized fashion. Example PARS and chemical-H&E whole-slide images are available at the S-BIAD2324 Archive. **Results:** Diagnostic sensitivity and specificity were comparable between PARS (sensitivity: 96.7% [95% CI: 89.5–100.0%]; specificity: 95.2% [95% CI: 74.7–100.0%]) and H&E (sensitivity: 95.6% [95% CI: 88.2–100.0%]; specificity: 85.7% [95% CI: 50.2–100.0%]). Primary diagnostic agreement was 95.5% (95% CI: 89.5–100.0%; Cohen’s κ=0.929 [95% CI: 0.869–0.989]), with high inter-rater reliability (Fleiss’ κ=0.886 [95% CI: 0.751–1.00] for PARS; κ=0.805 [95% CI: 0.652–0.958] for H&E). Inter-modality subtyping agreement was 91.8% (95% CI: 81.4–100.0%; Cohen’s κ=0.731 [95% CI: 0.375–1.000]) for SCC and 100% (κ=1.000) for BCC. For margin confinement, agreement was 92.2% (95% CI: 85.0–99.4%; Cohen’s κ=0.718 [95% CI: 0.452–0.984]). Dermatopathologists could not reliably distinguish image origin, and diagnostic confidence was statistically equivalent between modalities. **Conclusions:** Diagnostic performance of PARS virtual H&E images was consistent with established clinical benchmarks, providing encouraging preliminary evidence of diagnostic concordance with conventional H&E staining. While larger validation cohorts are required to establish true clinical equivalence, these initial findings highlight PARS label-free virtual histology as a promising approach to enhance dermatopathology diagnostics by preserving tissue for downstream analysis while supporting AI integration.

## 1. Introduction

Skin biopsies are a common surgical procedure performed when a suspicious skin lesion is identified (e.g., lesions with high-risk features of irregular borders, rapid growth or ulceration/bleeding), or to confirm a benign diagnosis for a lesion that requires resection due to local irritation or functional impairment. During a biopsy, part or all of the lesion is surgically removed [1,2]. Excised tissues are then assessed through histopathological methods to confirm or rule out malignancy [1,2,3,4]. For cancerous lesions, a margin assessment is also performed to identify the adequacy of resection [3,4,5]. Tissue margins are typically characterized as negative margins (edge of sample uninvolved with malignancy) or a positive margin where cancer is suspected to be left behind on the patient [3,4,5]. Both the histology and margins are important for guiding further management. For example, basal cell carcinomas with a positive margin could be safely monitored, whereas deeply invasive melanomas may require further oncologic resection locally, as well as imaging and sentinel lymph node assessment [3,4,5]. Subsequently, the skin biopsy process may typically result in a definitive diagnosis, while achieving negative margins (complete removal) for smaller lesions, serving both diagnostic and therapeutic purposes [3,4,5].

To enable histological analysis, skin excision samples undergo a standard tissue processing and staining procedure. Specimens are fixed in formalin, dehydrated, and embedded into paraffin wax. Embedded tissues are cut into thin translucent sections, placed onto slides, and stained with hematoxylin and eosin dyes (H&E) [6], the gold-standard stain for histological evaluation. Hematoxylin stains cell nuclei in blue-purple, while eosin stains extracellular and cytoplasmic components in shades of pink-red [6]. This cellular microanatomy is critical for distinguishing malignant and benign skin conditions, as well as to determining the type and grade of cancer where applicable [3,4,5,7].

Despite the indispensable diagnostic value, conventional tissue processing and labeling impart certain operational burdens. Chemical processing and staining are time-intensive, carry significant recurring costs, and require technical expertise [6,8,9]. Chemical labeling also imparts critical diagnostic limitations since current staining processes alter specimens’ chemistry. As a direct result, sequential staining or “re-staining” of slides is normally prohibited in clinical diagnostic settings. Producing secondary staining contrasts beyond H&E (e.g., Toluidine Blue, Periodic Acid Schiff, Jones Silver) or repeating an H&E stain which fails to meet diagnostic standards (due to inter-sample variability factors such as section thickness, staining protocols, and reagent quality) [6,8,9], requires a new tissue section. Repeated sectioning directly constrains the diagnostic utility of specimens, especially when there are small tissue volumes (e.g., core biopsy samples). In the worst case, there may be a need to perform a re-biopsy to collect additional samples for staining.

To address these constraints, there is growing interest in two categories of advanced label-free optical imaging technologies that may enhance the diagnostic workflow or compliment clinical pathways pre- and post-excision. The first category is non-invasive in vivo imaging technologies focused on real-time “virtual biopsies”. These methods aim to assess lesions and guide surgical margins prior to or during tissue excision. In clinical dermatology, modalities such as reflectance confocal microscopy (RCM) [10,11,12], and optical coherence tomography (OCT) [13,14], including variants like dynamic-OCT (D-OCT) [15,16] and line-field confocal OCT (LC-OCT) [17,18], have shown promise for intra-intraoperative evaluation. These techniques leverage optical scattering variations to capture tissues subsurface microstructures across planes (RCM) or in tissue volumes (OCT) [10,11,12,13,14]. These methods produce grayscale representations of scattering structures, and vasculature which can assist in lesion mapping and surgical planning to ensure the full margin is resected accurately the first time [10,11,12,13,14].

The second category focuses on non-destructive ex vivo imaging of resected tissue post-excision to maximize diagnostic utility and validate surgical findings. A key approach in this domain involves replicating traditional histochemical detail through the “virtual staining” of unprocessed specimens. Virtual staining applies deep learning image-to-image transforms to map label-free optical signals into familiar H&E color contrast. This pathway enables rapid diagnostic evaluation while preserving tissues for downstream ancillary testing, such as molecular profiling or immunohistochemistry. This can greatly enhance the diagnostic utility of each specimen, reducing the risk of re-biopsy.

Photon Absorption Remote Sensing (PARS) is an emerging label-free imaging platform which is well suited for this label-free imaging and virtual staining workflow [19,20,21,22]. PARS directly observes the dominant radiative (fluorescent) and non-radiative (thermal and pressure) relaxation signatures following an optical absorption event. This method uses a pump–probe design, where the pump excites specimens using a focused picosecond-scale pulse [19,20,21,22]. Then, radiative relaxation is measured as the emission of Stokes-shifted photons, while non-radiative relaxation is measured as transient fluctuations in the reflected or transmitted intensity of the co-focused probe beam. By simultaneously capturing both dominant de-excitation fractions, PARS provides unique insights into biomolecules’ excited state dynamics and directly detects key biological molecules, all without chemical dyes [19,20,21,22].

In label-free histological imaging PARS employs (266 nm) ultraviolet (UV) excitation to provide sensitivity to numerous diagnostic biomolecules, such as hemoglobin, DNA, collagen, elastin, and cytochromes [19,20,21,22,23,24]. The resulting PARS absorption data can subsequently identify a range of biomolecular features analogous to chemical staining. This forms a robust basis for deep learning-based PARS virtual staining. In practice, registered and paired PARS and chemically stained (e.g., H&E) whole-slide image datasets are used train image-to-image translation networks, such as Pix2Pix [25]. This process has been used to develop PARS-based virtual staining for a range of chemical stains, including H&E, PAS, Masson’s Trichrome, and Jones Silver [20,22,23,26,27]. Prior work has demonstrated that these PARS virtually stained images may be effectively indistinguishable from chemical staining within the same tissue samples [20,21,22,23,26]. For instance, one recent study of the PARS virtual H&E staining of breast core samples found significant pathologist agreement on diagnostic interpretation [21].

If translated into clinical workflows, PARS virtual histology subsequently offers capabilities which may enhance current diagnostic processes. PARS avoids key chemical staining limitations by capturing label-free endogenous biomolecular contrast. This means that PARS imaging does not chemically or mechanically alter specimens, unlike chemical staining. Following PARS scanning, each tissue specimen is completely preserved and can be reused for additional testing (e.g., immunohistochemical or secondary histochemical analysis). As a further advantage, the rich biomolecular-specific data captured in PARS typically holds more information than afforded by traditional chemical H&E staining. Hence, one PARS scan, completed within a similar time to brightfield slide scanning, can replicate several additional histochemical stains beyond H&E (e.g., PAS, Masson’s Trichrome, Jones Silver) [23,26,28]. Subsequently, PARS virtual histology may enhance the diagnostic utility of each tissue specimen by enabling (1) multiplexed virtual staining, and (2) sequential chemical staining of each tissue sample after PARS imaging. In turn, this may advance diagnostic timelines by circumventing chemical staining processes. Finally, as endogenous biomolecules are leveraged for contrast, PARS (and other similar techniques) has shown preliminary label-free imaging within bulk resected tissue specimens [29,30]. This has potential to circumvent the entire sample preparation workflow in the future.

In this prospective study, we focus on evaluating the diagnostic accuracy of the PARS virtual H&E imaged against gold-standard chemical H&E staining. This is a key validation step towards the ultimate goal of introducing PARS into clinical settings. This study specifically assesses diagnostic concordance between PARS virtual H&E staining and chemical H&E staining in whole-slide excisional skin biopsy samples. Sixteen whole-slide formalin-fixed paraffin-embedded (FFPE) skin excision samples were analyzed, representing normal skin, basal cell carcinoma (BCC), and squamous cell carcinoma (SCC). Unstained specimens were scanned using PARS to produce virtual H&E images. The same slides were then chemically H&E-stained and imaged using a 40× digital pathology scanner, producing a total set of 32 whole-slide images (16 PARS virtual H&E and 16 chemical H&E). For each primary diagnostic included in this study (SCC, BCC, benign), a matched PARS virtual and chemical H&E example are publicly available at the BioImage Archive [31] (see Data Availability). Seven fellowship-trained dermatopathologists independently reviewed these whole-slide images in a masked fashion and filled in diagnostic surveys. Concordance analysis was performed to quantify the diagnostic agreement, malignancy confinement, and diagnostic confidence between the PARS virtual H&E images and the gold-standard H&E-stained samples.

## 2. Materials and Methods

### 2.1. Sample Selection

Samples for this study were specifically gathered from archival FFPE tissue block samples, which were no longer necessary for patient diagnostics. Inclusion criteria specified primary human skin excisions with confirmed ground-truth diagnoses of non-melanoma skin cancer (basal cell carcinoma [BCC] or squamous cell carcinoma [SCC]) or normal/benign skin tissue. In addition, specimens were only selected if they presented sufficient volume for sectioning and had the section integrity necessary for paired imaging. Exclusion criteria were defined to eliminate cases outside the primary scope of the diagnostic survey. Excluded samples included pigmented malignancies (melanoma), infectious lesions (e.g., fungal dermatoses), inflammatory skin conditions, and severe tissue artifacts. Small tissue volumes, and redundant samples (e.g., repeat samples from the same excision) were also excluded.

Based on these criteria, a total of 21 independent skin excision cases were initially screened from archival formalin-fixed paraffin-embedded (FFPE) tissue blocks. From the screened selection, a representative subset of 16 FFPE tissue specimens was chosen to ensure balanced coverage across primary diagnostic categories and histological subtypes. The final set included five BCC cases, eight SCC cases, and three normal or benign skin samples.

### 2.2. Patient Materials and Ethical Considerations

The skin tissues were procured through collaboration with clinical partners. As indicated previously, samples were from archival tissues, with all patient identifiers and all sample information removed. All human tissue experiments were conducted in accordance with the government of Canada TCPS2 guidelines. Specific study protocols were approved by the University of Waterloo Health Research Ethics Committee (Protocol ID: 40275; Photon Absorption Remote Sensing Microscopy of Surgical Resection, Needle Biopsy, and Pathology Specimens). As samples were fully anonymized archival tissues, the ethics committees waived informed consent.

### 2.3. Sample Preparation Prior to PARS Imaging and Gold-Standard H&E Staining

Skin lesions were surgically excised from patients and immediately fixed in 10% neutral buffered formalin for 24 h. After fixation, a histotechnician prepared thin sectioned histopathology slides using a standard tissue processing and embedding workflow [6]. Specimens were dehydrated through graded ethanol rinses, cleared with xylene baths to remove ethanol and residual fats, and embedded into paraffin wax to form formalin-fixed paraffin-embedded (FFPE) tissue blocks. The FFPE blocks were sectioned with a microtome into ~4–5 μm translucent tissue sections, which were mounted on glass microscope slides and briefly heated to 60 °C to ensure adhesion and surface uniformity.

### 2.4. PARS Microscope Imaging

Label-free PARS images were acquired from the unstained tissue sections using a custom-built transmission-mode PARS microscope system (Waterloo, ON, Canada). Detailed descriptions of the optical system and imaging protocol are provided in a recent report by Tweel et al. [20]. In brief, PARS imaging was performed using a 50 kHz 400 ps 266 nm UV laser (Wedge XF 266, RPMC; Bright Solutions, Pavia, Italy) to excite biomolecules. Following each excitation, three main features were measured, representing the major absorption and scattering properties: radiative (fluorescence) emission, non-radiative heating (thermal/pressure effects), and local scattering.

To quantify the radiative signal intensity, emitted Stokes-shifted photons were spectrally isolated and measured with an avalanche photodiode (APD130A2; Thorlabs, Newton, NJ, USA). The radiative intensity value was recorded as the peak emission amplitude. To measure the scattering and non-radiative relaxation effects, a 405 nm continuous-wave probe beam was co-aligned with the excitation (OBIS-LS405; Coherent, Santa Clara, CA, USA). Non-radiative (photothermal and photoacoustic) signals were measured by observing heat- and pressure-induced modulations in the transmitted probe beam intensity following excitation. The time-resolved non-radiative modulation was measured by integrating the modulation to determine a percentage change in probe transmission. Scattering signals were recorded by measuring the 405 nm probe transmission intensity prior to excitation (APD130A2; Thorlabs, Newton, NJ, USA).

The three optical contrasts from each excitation event then correspond to one co-registered pixel in the final image. To form images, three-axis mechanical stages moved the sample across the objective lenses in a raster (“s”-shaped) scanning pattern. Pixels or excitation events were spaced 250 nm apart, achieving 40× digital imaging magnification. Matching optical resolution was provided using a 0.42 numerical aperture (NA) UV objective lens (NPAL-50-UV-YSTF; OptoSigma, Santa Ana, CA, USA) to focus both excitation and detection beams, while optical signals (probe light and radiative photons) were collected using a 0.7 NA objective lens (278-806-3; Mitutoyo, Aurora, IL, USA).

Whole-slide images were formed by discretizing the ROI into 500 × 500 μm sections which were independently scanned using the highlighted method. At each section, an autofocusing operation was performed prior to scanning. Section data was then contrast-matched and stitched together, forming a single whole-slide image, using the pipeline outlined in by Tweel et al. [22]. The result was a complete whole-slide image based on native tissue absorption characteristics without the need for exogenous labels.

### 2.5. Gold-Standard H&E Staining and Digital Image Acquisition

Following PARS imaging, all tissues samples underwent standard chemical H&E staining using standard histopathology protocols. Stained slides were digitized at 40× magnification using a transmission brightfield slide scanner (Morpholens 1; Morphle Digital Pathology, New York, NY, USA). This produced high-resolution images of the chemically stained tissue to serve as a ground truth for both training and validation.

The availability of precisely aligned virtual and chemical H&E images enabled robust image-to-image model development and direct head-to-head diagnostic comparison. The use of the same slide for both modalities (PARS prior to staining and H&E afterward) ensured matched cellular and architectural features, minimizing confounding due to sectioning artifacts or tissue heterogeneity. This method of matched image generation has been validated in previous studies [19,20,21,22,32].

### 2.6. PARS Virtual H&E Colourization

A conditional generative adversarial network, Pix2Pix [25], was trained to convert PARS label-free data into virtual H&E images. This approach differs from the previous PARS virtual staining validation study, which used a cycle-consistent generative adversarial network (CycleGAN) [21,33,34]. CycleGAN does not require registration between the ground truth, and target domain [21,33,34]. As a result, CycleGAN may provide more optimal virtual staining performance (e.g., superior image clarity and sharpness) in samples where it is difficult to provide pixel-level ground truth (such as the previously explored need-core biopsy samples) [21,25,33,34]. However, if pixel-level registration is possible, Pix2Pix can provide more robust correlations between structure and color, facilitating an improved domain transformation and better virtual staining image quality [25,33]. The key challenge to date has been developing a sufficient volume of pixel-level registered whole-slide image data for model training. To this end, this work utilizes a new WSI registration workflow, based on the Warpy framework [35]. This enables pixel-level whole-slide registration, facilitating successful implementation of the Pix2Pix virtual staining models.

**Figure 1:**
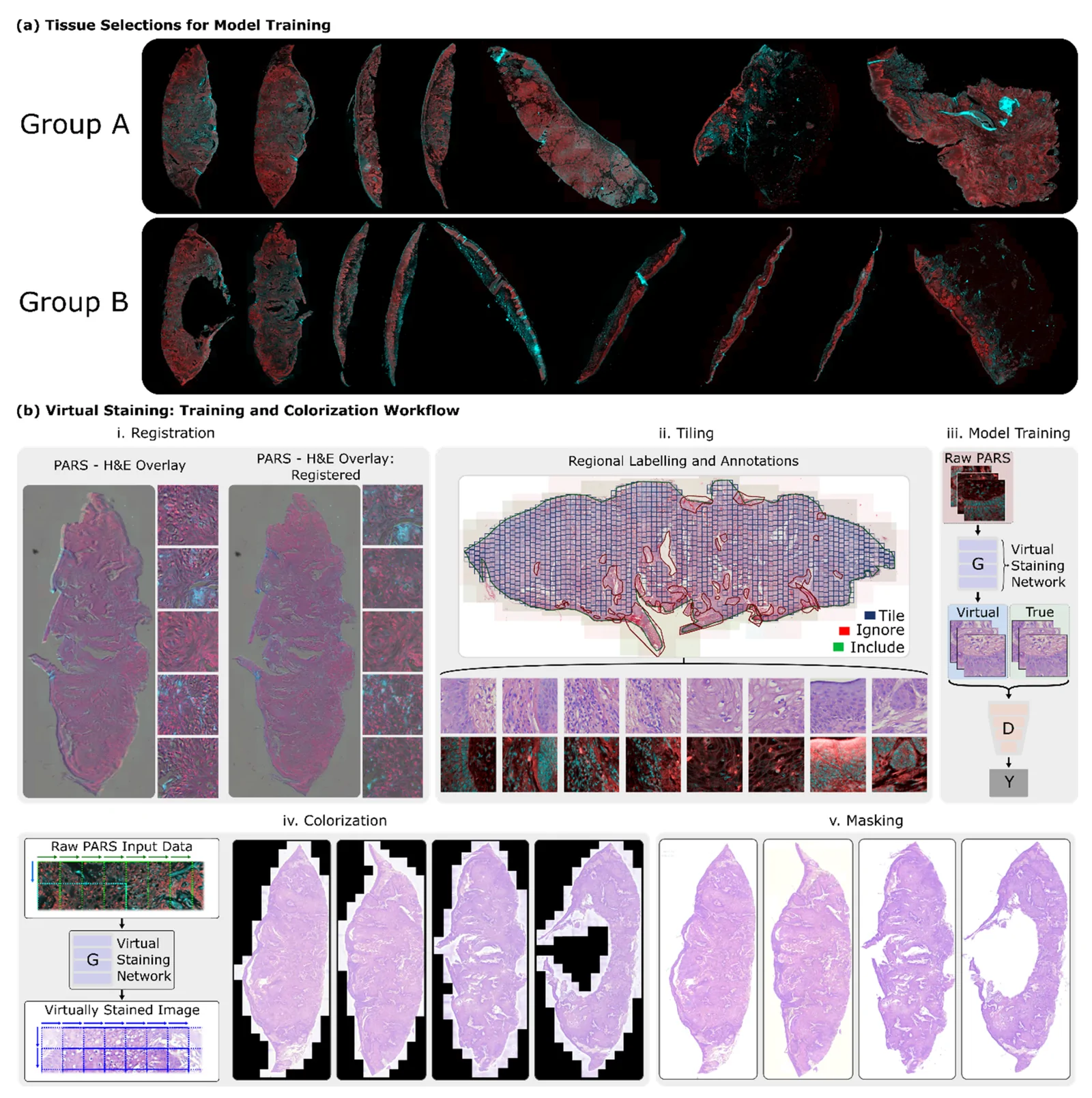
Workflow for developing PARS virtual H&E images. (**a**) Division of data for network training and colorization. Model (1) excluded data from Group A for training, while Model (2) excluded data from Group B during training. Models were then applied to virtually stain the respective groups ensuring only unseen data was colorized. (**b**) Steps performed to train and colorize tissues, including (**i**) whole-slide registration using the Warpy whole-slide registration workflow [33], (**ii**) data labeling and (512 × 512 pixel) training pair generation, (**iii**) algorithm training, (**iv**) colorization or model application using 75% linearly blended overlapping regions, and (**v**) masking to conceal unscanned areas.

In this study, two Pix2Pix virtual staining models were trained and applied to colorize the 16 whole-slide virtual H&E images. Model 1 excluded Group A slides during training and was applied only to Group A; Model 2 excluded Group B and was applied to Group B (Figure 1a). This ensured that colorization was performed only on unseen data, supporting robust validation.

Preparing the two Pix2Pix virtual staining models and developing the virtually stained images involved the following key steps (Figure 1b): (i) registration, (ii) tiling, (iii) training, (iv) colorization, and (v) masking. Whole-slide images (H&E and PARS) were registered using the Warpy workflow, which features manual rigid registration, automated affine registration, and a patchwise (500 × 500 µm patches) spline registration [35]. An example of slide overlays before and after registration are shown in Figure 1(bi). Registered whole-slide images were annotated to identify the tissue and exclude artifacts such as out-of-focus regions or unwanted features such as dust. Notably, surgical margin dye was also annotated and excluded from training sets. PARS optical absorption contrast can directly measure and display margin dye due to its strong absorption characteristics. However, ink-bearing regions were intentionally masked in this study to avoid replicating distinct inking patterns, which may cause reviewers to recognize image pairs.

Annotations were used to dice images into 512 × 512-pixel (128 × 128 μm) tile pairs (Figure 1(bii)) for model training. Model (1) used a total of eight slides (11,459 training pairs), while Model (2) used a total of seven slides (11,232 training pairs). Training was performed over 200 epochs, on a Nvidia RTX A6000 (Nvidia, Santa Clara, CA, USA) using pytorch 2.3.1 [36] and Cuda 11.8 (Nvidia, Santa Clara, CA, USA) (Figure 1(biii)). Model (1) was applied to virtually stain the 7 unseen slides of Group (A), while Model (2) was applied to the 9 unseen slides from Group (B), resulting in 16 colorized whole-slide images (Figure 1(biv)). During the colorization, the model was applied to overlapping 512 × 512-pixel patches, which were linearly blended. Both PARS virtual H&E, and the ground-truth H&E images were then masked (Figure 1(bv)) to hide unscanned regions and remove background features (e.g., dust), which could indicate the image origin.

### 2.7. Evaluation by Expert Pathologists

Seven fellowship-trained dermatopathologists, six with no prior exposure to PARS virtual histology images, independently reviewed the 32 images (16 PARS and 16 H&E) using a customized web-based histopathology platform (Waterloo, ON, Canada). Reviewers could freely navigate and zoom (up to 40×) each slide using the web interface. A pathologist scored each image on the parameters shown in Table 1.

Images were anonymized, randomly rotated, and assigned alphanumeric reference labels of AA through BF for survey purposes. All clinicopathologic details of the cases and the origin of the digital images (either true chemical H&E or PARS virtual H&E) were masked. Slides were presented in the following algorithmically randomized order: T16, T13, P8, T2, P4, P9, T14, P3, T6, T15, T12, P5, T11, P7, T1, T3, T10, P14, T8, T4, P11, T9, P6, P12, T7, P10, P15, P16, P13, P1, T5, P2. The ‘P’ prefix corresponds to PARS virtual H&E, while the ‘T’ prefix corresponds to true chemical H&E. This presentation sequence was designed using an algorithm which randomized image order while ensuring independent PARS and true H&E images from the same tissue were presented at least seven images apart. The aim was to minimize potential for biases within each image pair, which might arise if (for example) an image pair from the same tissue was shown back-to-back.

### 2.8. Statistical Analysis

Agreement between PARS and H&E diagnoses was primarily quantified using Cohen’s kappa (κ) (for intra-rater modality comparison) and Fleiss’ kappa (κ) (for inter-rater agreement within each modality). The Cohen’s kappa coefficient measures inter-rater reliability while accounting for the possibility of agreement occurring by chance [37]. This is key to assessing if two raters agree beyond what would be expected by chance alone. The Kappa assessment also provides robustness to varied prevalences in different data categories [37]. The Fleiss’ kappa measures the same characteristic as the Cohen’s kappa but compares more than two raters [38]. The resulting kappa values range from −1 to 1. Positive values indicate agreement beyond chance (1 indicates perfect agreement), while negative values indicate agreement worse than chance. Kappa values were interpreted as “fair” or “poor” for κ<0.6, “substantial” or “good” for κ=0.6–0.8, “near perfect” for κ>0.8 [37,38,39,40]. Percent agreement metrics aggregated at the sample or rater level were also reported alongside kappa scores to contextualize the findings.

Diagnostic performance for binary malignancy detection (malignant vs. benign) was evaluated using sample-aggregated sensitivity and specificity [41]. Pathologists’ ability to distinguish image origin (PARS vs. H&E) was evaluated against a 50% random chance baseline using a two-tailed exact binomial test [42]. To assess directional preference in cases where raters assigned different confidence levels or diagnoses to matched image pairs, a two-tailed exact Sign test was performed [42]. Differences in overall diagnostic confidence distributions between PARS and H&E were evaluated using a Chi-square test of independence ($\chi^{2}$) [42]. Continuous paired sample-level metrics between PARS and H&E modalities were compared using a paired t-test [42].

Two-sided 95% confidence intervals (95% CI) were calculated for all percentage agreements, sensitivity, specificity, and kappa coefficients. Confidence intervals were calculated as sample-level or rater-level metrics using Student’s t-distribution to account for sample size. All statistical analyses and inter-rater reliability calculations were performed using Python (v3.10; using scipy.stats [43] and irrCAC packages (v0.4.4) [44,45]) and R statistical software (version 4.2.0) [46].

## 3. Results

### 3.1. Example Whole-Slide Image Pairs

**Figure 2:**
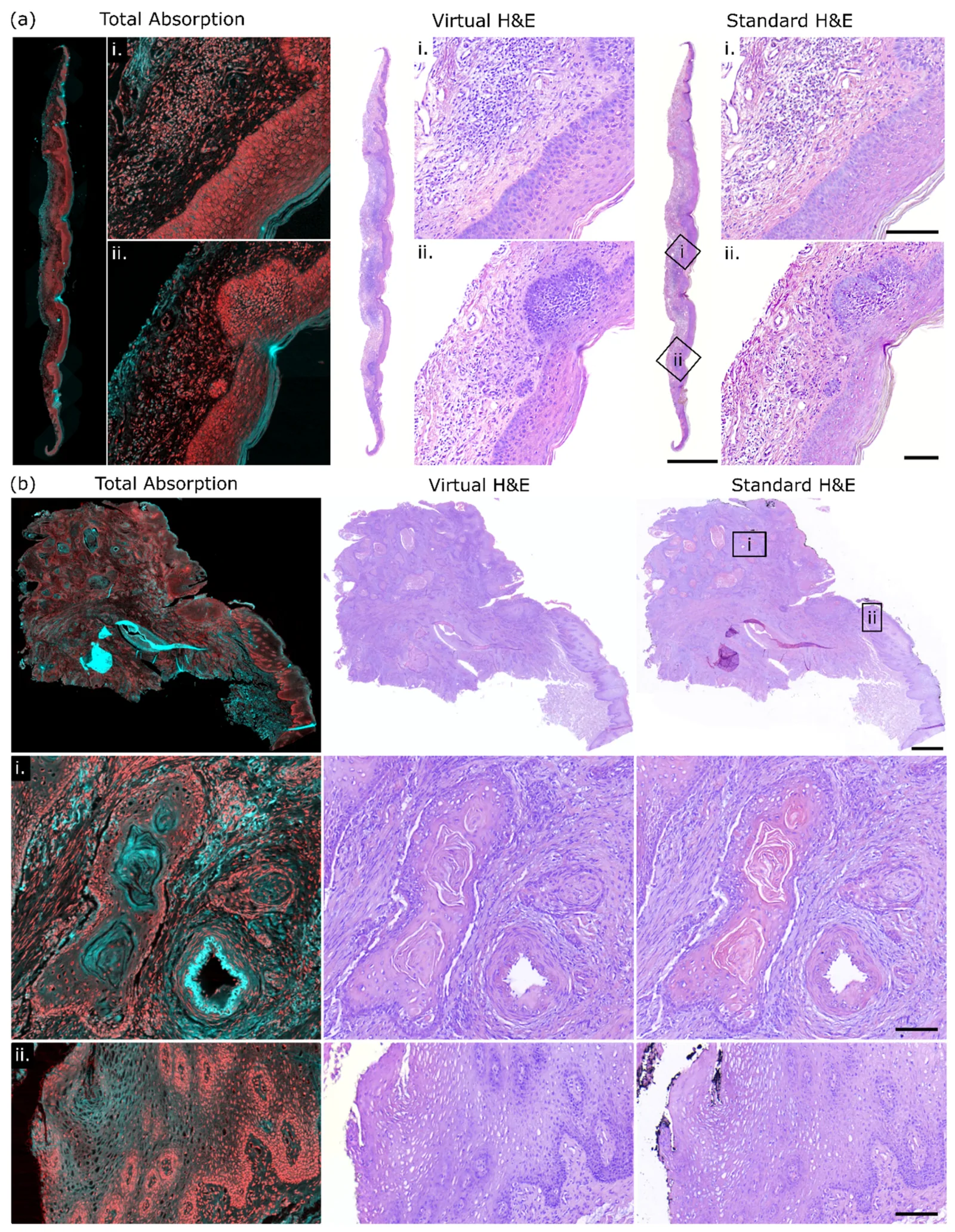
Examples of the PARS total absorption (TA), virtual H&E, and true H&E pairs used in this study. The samples exhibit closely matched staining colors and tissue morphology between the virtual and real representations. (**a**) Example of basal cell carcinoma (BCC). (**b**) Example of squamous cell carcinoma (SCC). Letters (i,ii) depict two regions of higher magnification on the sample. The same regions from all three contrasts are shown in the (i) and (ii) labelled figures corresponding to the sections annotated on the H&E images in (**a**) Standard H&E and (**b**) Standard H&E. (**a**,**b**) Scale Bar: 1 mm; (i,ii) Scale Bar: 100 μm.

Representative whole-slide images from two skin excision specimens are shown in Figure 2. For each tissue sample, the raw total absorption (TA) PARS image is presented alongside the PARS virtual H&E and real H&E image. In the TA PARS image, the radiative relaxation is colored in blue, while the non-radiative relaxation is colored red. This image represents the raw PARS data which is inputted into the virtual staining algorithm. The virtual and chemical H&E images represent subsections of the diagnostic views shown to pathologists during assessment. Unfortunately, it is challenging to illustrate the virtual staining performance through these subsection examples due to the spatial limitations. Hence, a subset of the of whole-slide PARS and chemical H&E images have been made publicly available at the BioImage Archive (see Data Availability). Slides are a standard OME-TIFF format and can be viewed and compared with standard pathology viewing software such as QuPath (>v0.5.0) [47].

Within the presented examples, Figure 2a illustrates a case of basal cell carcinoma (BCC). This sample exhibits basaloid nests arising from the epidermis and within the dermis. High-magnification views (regions i and ii in Figure 2a) reveal classic basaloid nests and cords with hyperchromatic nuclei, scant cytoplasm, and peripheral nuclear palisading. These tumor clusters are embedded in a mucinous or fibroblastic stroma, consistent with nodular or superficial BCC. The absence of keratinization distinguishes BCC from squamous cell carcinoma, where mitotic figures are common but rarely atypical.

Figure 2b depicts squamous cell carcinoma (SCC), arising from the epidermis and growing downward into the dermis. Higher magnification (regions i and ii in Figure 2b) shows atypical keratinocytes with pleomorphic, hyperchromatic nuclei and prominent keratinization. Features include dyskeratotic cells, keratin pearls, and intercellular bridges—all hallmarks of squamous differentiation. SCC invades the dermis in sheets, cords, or nests, with variable differentiation. Well-differentiated tumors show organized keratinization, while poorly differentiated lesions lack keratin and exhibit marked nuclear atypia and mitotic activity. Desmoplastic stroma may surround invasive nests, especially in aggressive variants.

Comparing the different visualizations, the “Total-Absorption” (TA) PARS images provide a clear basis for the success of the virtual staining. The deep UV excitation used in PARS is broadly absorbed in tissues, where the unique distribution of relaxation effects (radiative vs. non-radiative) provides specificity to critical diagnostic features. The non-radiative contrast (red) predominately highlights DNA which exhibits strong non-radiative relaxation, corresponding to similar features as hematoxylin staining. Conversely, the connective tissues (primarily collagen and elastin) exhibit appreciable radiative relaxation (blue), approximating eosin’s affinity for cytoplasm and stroma [19,20,21]. In some instances, PARS may reveal additional features which are not visualized by the H&E staining. For example, the inner elastic wall of an artery exhibits prominent radiative signal (region i of TA in Figure 2) not captured in standard H&E due to limited sensitivity to certain biomolecules. The virtual staining model suppresses these features to match the chemical H&E fidelity.

An additional advantage of the PARS virtual staining is its contrast consistency. In PARS, the signal (and virtual H&E stain contrast) is derived from the endogenous absorption. Hence, the virtual staining exhibits uniformity across all samples. In contrast, chemical H&E staining is subject to variability from factors such as section thickness, dye concentration, staining duration, specimen age, and storage conditions [48,49,50,51,52]. This improved consistency with virtual staining may support improved reproducibility in digital workflows.

As a further extension, while this work focuses only on virtual H&E staining, there are numerous other stains used in clinical settings. As an example, virtual staining networks could be trained to develop Periodic Acid Schiff and Toluidine Blue, which are used in certain cases of basaloid carcinomas [53,54]. In this work, H&E was selected to align with current practices for skin malignancy diagnosis. The turnaround for a pathology report is typically a week or more due to tissue preparation, chemical H&E staining, brightfield imaging, and human diagnostic interpretation. Hence, the additional stains beyond H&E are often avoided to mitigate further delays. However, each PARS scan of a single unstained slide generates rich, multidimensional molecular data that has the potential to replicate several chemical staining patterns. With the development of additional virtual staining models. Multiple stains may be developed from each tissue specimen. In addition, label-free imaging retains samples’ ancillary testing and analysis.

### 3.2. Primary Diagnosis

Pathologists were first asked to assign a primary diagnosis for each image (16 PARS and 16 H&E), resulting in 32 evaluations per rater for a total of 224 primary diagnoses. The corresponding primary diagnoses for PARS and H&E images are presented in Table 2. For reference, the expected consensus diagnosis for each specimen was established based on the most prevalent diagnosis across raters. While we acknowledge that basing our definition of ‘true diagnosis’ on the observed data could create statistical concerns if there was not a clear agreement across multiple raters, in our sample there was a unanimous agreement between raters in 12 of the 16 cases, unanimous bar one rater in 1 case, unanimous bar two raters in 2 cases, and just one case where there was a nearly even split. For the nearly even split, the diagnosis was confirmed during post-analysis through a consensus diagnoses.

Diagnostic performance was initially assessed at a binary level (i.e., Malignant vs. Benign samples) to determine the sensitivity and specificity of malignancy detection. This is a critical metric in clinical diagnostics to ensure malignant lesions are not missed. For malignancy detection, PARS achieved a sensitivity of 96.7% (95% CI: 89.5–100.0%; n=16 specimens) and a specificity of 95.2% (95% CI: 74.7–100.0%; n=16 specimens). H&E achieved 95.6% sensitivity (95% CI: 88.2–100.0%; n=16 specimens) and 85.7% specificity (95% CI: 50.2–100.0%; *n* = 16 specimens).

Pathologist responses for primary diagnosis (i.e., SCC, BCC, melanoma, benign) were then aggregated by sample for each modality to assess intra-modality inter-rater agreement. Overall agreement was high within both modalities. Diagnostic accuracy was 95.5% (95% CI: 89.5–100.0%; *n* = 16 specimens) with near-perfect inter-rater agreement (Fleiss’ κ=0.886; 95% CI: 0.751–1.00; n=16 specimens) for PARS images. Accuracy was 92.9% (95% CI: 86.0–99.7%; n=16 specimens) with high inter-rater agreement (Fleiss’ κ=0.805;95% CI: 0.652–0.958; n=16 specimens) for H&E images.

Of the 224 total primary diagnoses 13 individual evaluation-level errors occurred relative to ground-truth consensus (5 on PARS images and 8 on H&E images) (Table 2). Notably, 6 of these 13 discrepancies occurred on a single, difficult-to-diagnose specimen (Specimen 1: 3 Benign vs. 4 BCC on PARS, and 3 Benign vs. 4 BCC on H&E). Excluding this difficult-to-diagnose sample, primary diagnostic accuracy rose to 98.1% (95% CI: 95.3–100.0%; *n* = 15 specimens) for PARS images and 95.2% (95% CI: 90.4–100.0%; *n* = 15 specimens) for H&E images. While we acknowledge this analysis is being conducted post hoc based on the observed data, we include it to highlight the potential impact of this single difficult image on the results.

Inter-modality agreement between PARS and H&E was similarly strong (Table 3). Across raters, the average diagnostic concordance between PARS and H&E was 95.5% (95% CI: 90.2–100.0%; n=16 specimens), with a Cohen’s kappa of κ=0.929 (95% CI: 0.869–0.989; *n* = 16 specimens), indicating near-perfect inter-modality agreement. Across all evaluations, basal cell carcinoma (‘basal’) was selected 63 times, non-malignant (‘benign’) 45 times, squamous cell carcinoma (‘squamous’) 113 times, and melanoma 3 times. There were only five diagnostic discrepancies between matching PARS and H&E image pairs. Three pathologists achieved perfect inter-modality agreement (κ = 1.000), while the remaining four achieved near-perfect agreement κ = 0.800–0.900. These results indicate reliable and equivalent diagnostic discrimination between BCC, SCC, and benign lesions across modalities for the specimens evaluated.

### 3.3. Evaluation of Cancer Subtypes and Grading

Following primary diagnostic identification, secondary evaluations were conducted to assess concordance in histological differentiation grading and risk subtyping among identified malignancies (as defined in Table 1). The objective was to evaluate virtual H&E images on a challenging diagnostic task (i.e., histological subtyping and grading). In the dermatopathology literature, cancer subtyping and grading (particularly for squamous cell carcinoma) are well-established as complex tasks, exhibiting substantial subjective inter-rater variability even on conventional H&E slides [55,56,57,58].

#### 3.3.1. Squamous Cell Carcinoma Grading

To evaluate histological differentiation grading among SCC cases (in situ, well-differentiated, moderately differentiated, or poorly differentiated SCC), inter-rater agreement was measured across specimens where all seven pathologists unanimously agreed on a primary diagnosis of SCC. In total, SCC was diagnosed 113 times across all evaluations (an odd number due to a single primary diagnosis discrepancy between PARS and H&E). From these 113 diagnoses, there were 7 consensus specimens (14 total images: 7 PARS and 7 H&E) with complete primary diagnostic agreement across all seven raters.

Inter-rater agreement for SCC grading across these seven consensus specimens demonstrated poor inter-rater concordance within both modalities: Fleiss’ κ=−0.012 (95% CI: −0.226 to 0.202; n=7 specimens) for H&E images and Fleiss’ κ=0.039 (95% CI: −0.190 to 0.269; n=7 specimens) for PARS images. This indicates negligible inter-rater agreement for SCC differentiation grading among pathologists within either modality, consistent with the established dermatopathology literature on conventional H&E slides.

To evaluate self-consistency, intra-rater inter-modality agreement between matched PARS and H&E images was assessed across all seven pathologists. In total, there were 56 matched image pairs where a pathologist assigned a primary diagnosis of SCC to both modalities (Table 4). Across these evaluations, the reported SCC grades were predominantly well-differentiated (n=68) or moderately differentiated (n=39), with poorly differentiated (n=3) and in situ (n=2) selected infrequently. Intra-rater concordance between matched H&E and PARS image pairs averaged 91.8% (95% CI: 81.4–100.0%; n=7 raters), with a mean Cohen’s κ of 0.731( 95% CI: 0.375–1.000; n=7 raters). This demonstrates that individual pathologists produced highly self-consistent subtype interpretations across virtual and chemical H&E images despite the low inter-rater agreement. This is again consistent with the established dermatopathology literature on conventional H&E slides. Notably, Rater 1 exhibited a low kappa (κ=0.000) despite achieving ~71.4% concordance (5/7 pairs), illustrating a well-documented artifact of the kappa statistic when a rater selects a single category with near-uniform frequency in small sample sizes.

#### 3.3.2. Basal Cell Carcinoma Subtyping

To evaluate histological grading of BCC cases (i.e., low vs. high), inter-rater agreement was assessed on specimens which expressed a primary diagnosis of BCC. In total BCC was selected as the primary diagnosis 63 times across both modalities (32 PARS, 31 H&E). From these 63 selections, there were three image pairs (three PARS images and three H&E images) where there was 100% agreement in the primary diagnosis of BCC.

Inter-rater subtyping agreement was assessed exclusively on these three tissue specimens where there was a unanimous primary diagnosis of BCC on both modalities (PARS and H&E). Across these three specimens, a total of 21 matched rater-image pairs were evaluated (42 total subtyping ratings: 21 PARS and 21 H&E). At the sample level (n=3 specimens), pathologists demonstrated high intra-modality inter-rater agreement within PARS images 95.2% (95% CI: 74.7–100.0%; Fleiss’ κ= −0.050, 95%CI:  −1 to 1, *n* = 3 samples) and within chemical H&E images 95.2% (95% CI: 74.7–100.0%; Fleiss’ κ=−0.050, 95% CI:  −1 to 1, n=3 samples). It is noted that the low Fleiss kappa and very broad confidence intervals are due to the high prevalence of agreement with few categories of selection. As this is a known effect with the Kappa statistic, it may be more appropriate to assess the raw percentage agreement for this comparison.

Inter-modality and intra-rater agreement was assessed using all instances, where pathologists evaluated BCC for both matched PARS and H&E images in a pair. Excluding five discordant evaluations where BCC was assigned to only one image in a matched pair (Table 3), 29 concordant rater-image pairs remained, resulting in 58 total risk subtyping evaluations (29 PARS and 29 H&E). Of these 58 evaluations, 56 were classified as low-risk BCC and 2 as high-risk BCC. Both high-risk selections were made by the same pathologist (Rater 4) on the exact same matched PARS and H&E specimen pair (Specimen 15). Consequently, PARS and H&E images demonstrated 100.0% inter-modality concordance (29/29 matched pairs; Cohen’s κ = 1.000) for BCC risk subtyping within concordant primary diagnostic evaluations.

#### 3.3.3. Melanoma Identification

Melanoma grading is not assessed as melanoma samples were not included within the dataset of this study. Melanoma was included in the diagnostic survey strictly as a distractor option to evaluate false-positive misclassification risk. In total, melanoma was selected only three times across all 224 primary diagnostic evaluations (Rater 1: two selections; Rater 6: one selection), with secondary subtype features reported in only one instance. Aggregated at the specimen level, this corresponds to a mean selection rate of 1.34% (95% CI: 0.00–3.41%; n=16 specimens). Due to the low frequency, a kappa value was not computed for these selections.

### 3.4. Evaluation of Cancer Margins/Tissue Edges

If pathologists identified malignancy (BCC, SCC, or melanoma), they were asked to assess histological resection margins as positive or negative. A tumor confined within the boundaries of the tissue specimen corresponds to negative margins, indicating a complete resection. Inter-rater agreement was initially evaluated across the 10 tissue specimens (10 PARS and 10 H&E images) with universal primary malignancy diagnosis across all seven raters. The remaining (n=6) specimens were either non-malignant (n=3) or had less than 100% agreement in primary diagnosis (n=3). Within this consensus set of 10 specimens, overall intra-modality inter-rater agreement was high for both H&E and PARS images, corresponding to a Fleiss’ κ=0.784 (95% CI: 0.321–1.000; n=10 specimens) for H&E images and Fleiss’ κ=0.824 (95% CI: 0.393–1.000; n=10 specimens) for PARS images.

Notably, in 9 of the 10 specimens there was unanimous agreement in the margin assessment. All margin assessment disagreements (within this 10-specimen consensus set) occurred on a single matched image pair (responses were 4 ‘yes’ [unconfined/positive margin]/3 ‘no’ [confined/negative margin] for H&E, and 6 ‘yes’/1 ‘no’ for PARS). The distribution of responses suggests that this specific specimen was difficult to assess, potentially due to section orientation or specimen quality. Excluding this single case, there was perfect (100.0%) inter-rater agreement (κ=1.000) for margin assessment within both PARS and H&E modalities (n=9 tissues).

Inter-modality intra-rater agreement was also evaluated to assess the consistency between matched PARS and H&E image pairs. In total, there were 86 matched pairs where a pathologist assigned a concordant primary diagnosis of malignancy across both modalities. This corresponded to a total of 178 recorded margin assessments (140 “confined” [negative margin] and 38 “unconfined” [positive margin]) across the seven raters. Evaluated across raters to prevent pseudoreplication, the average inter-modality agreement was 92.2% (95% CI: 85.0–99.4%; n=7 raters), with a mean Cohen’s κ=0.718 (95% CI: 0.452–0.984; n=7 raters). This indicates significant inter-modality concordance for surgical margin assessment between PARS virtual histology and conventional chemical H&E images (Table 5).

### 3.5. Image Origin

Raters were asked to classify image origin as chemical H&E, virtual (PARS), or uncertain (Table 6). The option “uncertain” was chosen frequently (86/224 evaluations; 38.4%), indicating pathologists were often unsure of image origin. Notably, 32 of these “uncertain” responses were attributed to a single respondent (Rater 4) who selected “uncertain” for all 16 PARS and 16 H&E images. Among the remaining raters, chemical H&E images were misidentified as virtual PARS in 25 instances, while virtual PARS images were misidentified as genuine chemical H&E in 38 instances.

Overall, raters were only slightly more likely to consider chemical H&E images genuine (40.2%; 95% CI: 9.8–70.6%; n=7 raters) than virtual PARS images (33.9%; 95% CI: 4.9–63.0%; n=7 raters; paired t-test t=1.449, p=0.197). Furthermore, when excluding “uncertain” responses, pathologists correctly identified image origin in only 54.3% of decisive evaluations (75/138), which is statistically indistinguishable from random chance (p=0.354, binomial test). These results demonstrate that pathologists could not reliably distinguish between conventional chemical H&E staining and PARS virtual H&E imaging, beyond the expectation of chance.

### 3.6. Diagnostic Confidence

Respondents were asked to rate their diagnostic confidence as Low, Moderate, or High (Table 7). Of 224 total responses, 167 were high, 42 moderate, and 15 low, distributed between the PARS and H&E data. The resulting distributions of confidence scores were found to be statistically indistinguishable between modalities (Chi-$SquareTest;\;{Χ}^{2}=0.168,\;p=0.919)$. Concurrently, the inter-rater agreement for overall diagnostic confidence was found to be fair within the PARS (Fleiss’ κ=0.235; 95% CI: −0.001 to 0.470; n=16 specimens), and within the H&E images (Fleiss’ κ=0.264; 95% CI: 0.069 to 0.460; n=16 specimens).

Inter-modality intra-rater agreement was also calculated to compare raters’ diagnostic confidence between matched image pairs. In total, raters had the same diagnostic confidence in 89/112 selections. Aggregated across samples, this corresponds to an average agreement of 79.5% (95% CI: 66.4–92.5%; n=16 specimens). In parallel, the average Cohen’s aggregated kappa across raters was 0.360 (95% CI: 0.037–0.682; n=7 pathologists) indicating fair agreement of diagnostic confidence between the PARS and H&E images.

As further assessment, instances where a rater did not find the same diagnostic confidence in a matched PARS and H&E pair were assessed. In total there were 23 cases (spread across the seven raters and 16 sample pairs) where diagnostic confidence differed within a PARS and H&E image pair. Of the disagreements, 11/23 favored the PARS image, while 12/23 favored the H&E images indicating no statistical preferences (Two-Tailed Sign Test, p=1.00).

## 4. Discussion

### 4.1. Study Summary and Key Findings

For detecting the presence of malignancy (e.g., Malignant vs. Benign) PARS demonstrated high concordance in sensitivity and specificity compared to conventional chemical H&E staining. PARS virtual H&E achieved a mean sensitivity of 96.7% (SD: 11.9%; 95% CI: 89.5–100.0%; n=13 malignant samples), closely matching the chemical H&E performance (95.6% (SD: 12.2%; 95% CI: 88.2–100.0%; n=13 malignant samples)). In benign tissues, PARS virtual H&E demonstrated a mean specificity of 95.2% (SD: 8.2%; 95% CI: 74.7–100.0%; n=3 benign samples), while chemical H&E yielded a mean specificity of 85.7% (SD: 14.3%; 95% CI: 50.2–100.0%; n=3 benign samples).

For the primary diagnosis, raters showed near perfect inter-rater agreement 95.5% (95% CI: 89.5–100.0%; *n* = 16 specimens) within PARS (Fleiss’ κ=0.886; 95% CI: 0.751–1.000; n=16 specimens) and 92.9% (95% CI: 86.0–99.7%; n=16 specimens) within H&E images (Fleiss’ κ=0.805; 95% CI: 0.652–0.958;n=16 specimens). Inter-modality agreement was also strong between the PARS and H&E images with a total 95.5% (95% CI: 90.2–100.0%; n=16 specimens) agreement (κ=0.929; 95% CI: 0.869–0.989; *n* = 16 specimens).

Substantial concordance was also achieved for malignancy subtype and resection margin assessment. For SCC subtyping, there was 91.8% inter-modality agreement (Cohen’s κ=0.731; 95% CI: 0.375–1.000) between PARS and H&E images, while BCC subtyping exhibited perfect agreement across all raters (100.0%; Cohen’s κ=1.000). When assessing resection margins, there was substantial intra-modality agreement within H&E (Fleiss’ κ=0.784; 95% CI: 0.321–1.000) and PARS (Fleiss’ κ=0.824; 95% CI: 0.393–1.000), alongside strong inter-modality agreement (92.2%; Cohen’s κ=0.718; 95% CI: 0.452–0.984).

Importantly, raters were unable to reliably distinguish virtual from real H&E images (54.3% accuracy; p=0.354), and diagnostic confidence was equivalent between modalities ($\chi^{2}=0.168,p=0.919$). In some measures, such as margin assessment (PARS Fleiss’ κ=0.824 vs. H&E Fleiss’ κ=0.784) or primary diagnosis (PARS Fleiss’ κ=0.886 vs. H&E Fleiss’ κ=0.805), PARS even slightly outperformed chemical H&E images. This suggests that PARS virtual staining may enable greater consistency in diagnosis compared to chemical H&E. These differences may reflect an improvement in stain standardization, as virtual staining reduces some of the staining related pre-analytic variability associated with chemical protocols (e.g., reagent quality, and staining duration) [6,8,9]. Overall, this analysis supports the conclusion that PARS virtual H&E images can provide a diagnostic performance matching conventional H&E images.

### 4.2. Error Analysis

As further insight, the primary diagnostic errors were analyzed to assess the potential source of diagnostic discrepancies in this study. Across all 224 evaluations (112 per modality), there were a total of 13 diagnostic errors. These errors occurred in five PARS evaluations and eight chemical H&E evaluations. False negatives accounted for (3/5) discordant diagnoses in PARS and (4/8) discordant diagnosis in H&E. Notably six (three H&E and three PARS) of the seven total false-negative responses were clustered on a single BCC specimen (Sample 1). This concentration of diagnosis indicates that the false-negative discrepancies were driven by morphologic subtleties and diagnostic complexity rather than limitations of the PARS virtual staining modality. False positives account for (1/5) discrepancies in PARS and (3/8) discrepancies in H&E. In PARS the false positive was an SCC misclassification. In chemical H&E there were two false-positive SCC misclassifications and one melanoma distractor selection. The remaining discordant diagnosis (1/5) in PARS and (1/8) in H&E involved a single SCC specimen (Sample 10), where a rater selected the melanoma distractor option on both modalities.

These sample-level metrics indicate that PARS label-free imaging may enable clinical diagnostics, which are directly comparable to chemical H&E staining at the malignancy detection and primary diagnosis level. This is further supported by the distractor group results in this survey. In this study melanoma was specifically omitted from the tissue set but was added to the survey as a distractor option. In total, melanoma was selected only 3 times (2 H&E and 1 PARS) across all 224 primary evaluations 1.34% selection rate (95% CI: 0.00–3.41%; n=16 specimens). As an additional confirmation step, post-analysis was performed on the images where melanoma was selected to ascertain the potential rationale behind the selection. For all three examples, post-analysis and pathologist consensus definitively ruled out the presence of melanoma features. However, sparse areas of keratinization and connective tissues were found to exhibit a more brown-yellowish tinge on certain monitors, as compared to other samples in this study. It is possible these features acted as a confounding factor during the primary diagnosis.

Overall, there is no presence of elevated false positives and no concerning error patterns in the PARS virtual histology diagnostics. This further confirms that PARS histology does not confound the diagnostic processes, supporting its diagnostic utility in dermatopathology.

### 4.3. Interpretation of Findings in Context

While chemical H&E staining remains the diagnostic gold standard, it is subject to interobserver variability and process-related inconsistencies. Developing a diagnosis often requires assessment of subtle features of nuclear morphology and tissue organization [3,4,59,60,61]. This complicates the evaluation of new histologic techniques, such as PARS virtual H&E images, since the “gold-standard” chemical H&E assessment is itself imperfect. In the context of this study, PARS virtual histology in skin excisions is shown to be diagnostically equivalent to chemical H&E staining assessed through digital histopathology.

Diagnostic concordance for common skin cancers such as BCC and SCC is generally high [5,7]. Well-defined and common diagnoses report agreement exceeding 90% in many cases [5,7]. This aligns with our findings for the primary diagnosis, which exhibited high inter-rater agreement (PARS: (Fleiss’κ=0.886; 95% CI: 0.751–1.000; n=16 specimens), H&E (Fleiss’ κ=0.805; 95% CI: 0.652–0.958;n=16 specimens)) and high diagnostic concordance (95.5% (95%CI: 90.2–100.0%; *n* = 16 specimens); κ = 0.929 (95% CI: 0.869–0.989; n=16 specimens)). However, performance can vary depending on the complexity or rarity of the lesion, and the pathologist’s specialization [59,60,61].

In this study, there was significant inter-modality agreement between the PARS and H&E (91.8% (95% CI: 81.4–100.0%; n=7 raters); Cohen’s κ=0.731 (95% CI: 0.375–1.000; n=7 raters)). for the SCC subtype, despite poor inter-rater agreement (H&E: κ=−0.012 (95% CI: −0.226 to 0.202; n=7 specimens), PARS: κ=0.039 (95% CI: −0.190 to 0.269; n=7 specimens)). Concurrently, the diagnostic confidence exhibited only fair inter-rater agreement (PARS: κ=0.235 (95% CI: −0.001 to 0.470; n=16 specimens), H&E: κ=0.264 (95% CI: 0.069 to 0.460; n=16 specimens)), with a nearly identical distribution of confidence scores across PARS and H&E. This indicates that despite the potential variability between individual pathologists’ interpretation, pathologists were generally self-consistent. This finding is in line with the dermatopathology literature. Certain tasks like cancer subtyping and grading (particularly for squamous cell carcinoma) are well established as highly pathologist-dependent [55,56,57,58,59,60,61]. These diagnostic tasks typically result in substantial subjective inter-rater variability even on conventional H&E slides [55,56,57,58,59,60,61].

To this end, variability remains a concern, especially for rare subtypes or lesions at the interface of benign and malignant features, despite high overall concordance among common skin cancers. This illustrates the importance of new methods and improvements to diagnostic tools, which may enhance reliability and accuracy. As an example, virtual staining could help to reduce pre-analytic variations. Unlike chemical staining, which is sensitive to section thickness, tissue processing, and reagent variability [6,8,9,50,51,52,53], PARS may have fewer potential sources of variability. By removing staining effects (e.g., reagent variability) the pre-analytic variables are limited to the factors which affect FFPE section quality (e.g., fixation time, embedding protocol, section thickness, sectioning quality). This partial standardization may allow for better color consistency across the PARS images, which in turn may help to explain the high inter-rater agreement observed for PARS across multiple diagnostic tasks. Coupled with emerging technologies in AI diagnostics, PARS has significant potential to enhance these diagnostic processes [62,63,64].

### 4.4. Strengths, Limitations, and Future Research

Careful consideration was taken to minimize factors which could unduly influence pathologist responses. Pathologists were carefully masked to tissue origin. Images were randomly oriented and randomly ordered, with an enforced minimum spacing between image pairs. Image backgrounds were removed to further mask the underlying modality. Similarly, while PARS inherently detects optical absorption from surgical margin inks, ink was intentionally excluded from the PARS virtual H&E images to prevent recognizable colored ink boundaries repeated between image pairs. In addition, every effort was made to ensure that standard diagnostic practices were facilitated. Pathologists were allowed to freely assess the 40× digital histology images as they would during conventional workflows. Examples of these whole-slide PARS and chemical H&E images are publicly available at the BioImage Archive (see Data Availability). Efforts were also taken to minimize confounding effects from sample variance. PARS and H&E images were derived from identical tissue sections, rather than adjacent slides, ensuring direct nuclei-to-nuclei pairing and identical diagnostic features in each image.

The conclusions presented here are encouraging but limited by the sample size of tissues (n=16 slides) and the pathologists (n=7  raters). Despite the relatively high number of training patches used to develop the deep-learning models, there were only 16 whole-slide images used for training. Hence, this work serves mostly to perform a preliminary validation of the virtual staining method. It is not intended to validate this specific model for clinical diagnostics across a wide scale. For this method to be accepted in clinical settings, these models must be trained and validated with orders of magnitude more independent samples than the limited set of 16 used in this study. Moreover, these models must be trained on a wider diagnostic scope as the current study limits the focus to the most common skin lesions (BCC and SCC).

To this end, future studies should expand the number of raters and increase the number of samples to further generalize and expand upon the findings presented here. Other studies should also expand the diagnostic scope to incorporate melanocytic lesions and additional cancer subtypes. These works should also expand outside of skin malignancies to include other major cancer types, such as gastrointestinal cancers.

In addition, future studies should include additional histochemical stains. This is an exciting avenue of development, given PARS’s unique ability to produce multiplexed virtual histochemical visualizations from a single scan [23,26,28]. This offers a direct avenue to improve the diagnostic accuracy beyond chemical staining workflows by allowing multiplexed virtual staining on each of the assessed specimens. Finally, future studies should focus on additional sample formats. While the present study focuses on FFPE specimens, PARS has previously shown the ability to image thick unstained specimens, including bulk unprocessed tissues [19,29]. Future works may explore using PARS to provide virtual staining (e.g., H&E) directly within bulk unprocessed tissues (freshly resected, or preserved).

## 5. Conclusions

PARS label-free virtual histology imaging offers a promising complementary approach to chemical staining workflows. By observing the dominant radiative (fluorescent) and non-radiative (thermal) relaxation processes following an optical absorption event, PARS generates rich datasets, which are a strong starting point for artificial intelligence imaging and diagnostic methods. This has enabled label-free PARS virtual staining, which is effectively indistinguishable from chemical staining, for a range of dyes (e.g., PAS, Masson’s Trichrome, Jones Silver) [26,28].

This study demonstrates that PARS-derived virtual H&E images achieve strong preliminary diagnostic concordance with chemical H&E staining across major metrics. Pathologists interpreted PARS images with similar confidence and accuracy and could not reliably distinguish image origin. These findings add to a growing body of work indicating that PARS virtual histology represents a viable candidate for histological evaluation. These findings simultaneously serve as an encouraging step towards validating PARS virtual H&E contrast, moving towards the goal of virtual H&E diagnostics [20,21,22]. In future, PARS virtual staining may significantly enhance the diagnostic utility of specimens by reducing tissue consumption, providing a multiplexed histology representation, and enabling ancillary diagnostic testing. This could eventually streamline histological workflows, reducing timelines and cost, while producing datasets which enable seamless integration towards AI diagnostics.

Future extensions of this work include larger multi-institutional validation studies, exploration of broader staining repertoires, and exploration of additional malignancies and tissue types. In addition, because PARS targets endogenous contrast of the tissues, this technique may be applied to other forms of tissue, including freshly resected specimens [19] and living tissues in vivo [65]. Consequently, future works may explore PARS virtual histology for imaging freshly resected tissues (e.g., Mohs surgical resections).

In summary, due to its high-fidelity virtual histology capabilities, PARS imaging stands to transform the landscape of digital pathology.

## Figures and Tables

**Table 1:** Survey questionnaire given to pathologists for each of the 32 images.

Question:	Answers:
1. The primary tissue diagnosis is:	☐ Non-Malignant/Benign ☐ Squamous Cell Carcinoma ☐ Basal Cell Carcinoma ☐ Melanoma
2. Squamous Cell Carcinoma	☐ In situ SCC only ☐ Well differentiated SCC ☐ Moderately differentiated SCC ☐ Poorly differentiated SCC
3. Basal Cell Carcinoma	☐ Low Risk * ☐ High Risk †
4. Melanoma	☐ High Dermal Mitotic Rate ☐ Skin Ulceration ☐ Desmoplastic melanoma ☐ Lymphatic invasion ☐ Brisk Tumor Infiltrating Lymphocytes
5. Malignancy confined within the boundaries of the tissue in this section/margins negative	☐ Yes ☐ No
6. This digital image comes from H&E	☐ Yes ☐ No ☐ Uncertain
7. My diagnostic confidence is	☐ Low ☐ Moderate ☐ High

* e.g., nodular, superficial, pigmented, infundibulocystic, fibroepithelial. † e.g., basosquamous, sclerosing/morpheaform, keloidal, infiltrative, sarcomatoid.

**Table 2:** Summary of pathologist responses to the statement “The primary tissue diagnosis is”. Note: the following short forms are used: Mel—Melanoma; Ben—Benign.

	PARS				H&E				
Diagnosis	SCC	BCC	Mel	Ben	SCC	BCC	Mel	Ben	Expectation
SCC	55	0	1	0	55	0	1	0	56
BCC	0	32	0	3	0	31	0	4	35
Melanoma	0	0	0	0	0	0	0	0	0
Benign	1	0	0	20	2	0	1	18	21
**Total**	56	32	1	23	57	31	2	22	112

**Table 3:** Summary of pathologist responses to statement “The primary tissue diagnosis is”.

		H&E				
	Diagnosis	SCC	BCC	Melanoma	Benign	Total
**PARS**	SCC	56	-	-	-	56
	BCC	-	30	-	2	32
	Melanoma	-	-	1	-	1
	Benign	1	1	1	20	23
	Total	57	31	2	22	112

**Table 4:** Summary of pathologist responses to the question of the Squamous Cell Carcinoma Subtyping. Results are aggregated across raters (n=7) with a 95% confidence interval: “[lower, upper]”.

Rater	Agreements	Total SCC Selections	Kappa
1	5	7	0.0
2	6	8	0.5
3	9	9	1
4	8	8	1
5	8	8	1
6	7	8	0.619
7	8	8	1
**Total**	51	56	0.73 [0.375, 1.00]

**Table 5:** Summary of pathologist responses to the question of malignancy confinement. Results are aggregated across raters (n=7) with a 95% confidence interval: “[lower, upper]”.

Rater	Agreements	Total Margin Selections	Kappa
1	10	10	1
2	11	13	0.409
3	12	14	0.44
4	13	13	1
5	12	12	1
6	10	12	0.429
7	11	12	0.75
**Total**	79	86	0.718 [0.452, 0.984]

**Table 6:** Summary of pathologist responses to statement “This digital image comes from H&E”.

		Rater							
Image	Response	1	2	3	4	5	6	7	Total
H&E	Genuine	10	0	9	0	11	12	3	45
	Uncertain	1	11	0	16	2	0	12	42
	Virtual	5	5	7	0	3	4	1	25
PARS	Genuine	10	0	6	0	7	13	2	38
	Uncertain	3	9	2	16	2	0	12	44
	Virtual	3	7	8	0	7	3	2	30

**Table 7:** Summary of pathologist responses to the statement “My diagnostic confidence is”. Results are aggregated across samples (n=16) with a 95% confidence interval: “[lower, upper]”.

Score	H&E	PARS	Total
Low	8	7	15
Moderate	20	22	42
High	84	83	167
Kappa	0.264 [0.069, 0.460]	0.235 [−0.001, 0.470]	

## Data Availability

A subset of representative whole-slide PARS and chemical H&E image pairs have been uploaded to the BioImage Archive. This data is publicly available under accession number S-BIAD2324, accessible at https://doi.org/10.6019/S-BIAD2324. Data is provided in OME-TIFF format and can be viewed with standard pathology software such as QuPath. Other datasets used and analyzed during this study are available from the corresponding author on reasonable request.
